# Cliniques at Rush College

**Published:** 1867-11

**Authors:** 


### *Cliniques at Rush College.*

A large number of capital operations have already been performed before the medical class at the College. At the last clinic, Prof. Gunn removed, from the bladder, ten distinct large calculi. The patient, a young man, 20 years of age, is doing well. It is expected that details will be given soon.

The advantages furnished by this city, for clinical teaching, are being taken possession of in earnest, and will be developed to their uttermost.

The Dispensary is open daily, for gratuitous treatment (both medical and surgical) of those whose circumstances will not permit large pecuniary expenditure.

